# Noise-Induced Hearing Loss in Police Officers: Systematic Review

**DOI:** 10.22038/IJORL.2022.64036.3198

**Published:** 2022-09

**Authors:** Marco Pollarolo, Angelo Immordino, Palmira Immordino, Federico Sireci, Francesco Lorusso, Francesco Dispenza

**Affiliations:** 1 *Department of Biomedicine, Neuroscience and Advanced Diagnostics, Azienda Ospedaliera Universitaria Policlinico ‘‘Paolo Giaccone’’, University of Palermo, Via del Vespro, 133, 90127 Palermo, Italy.*; 2 *Department of Health Promotion, Maternal and Infant Care, Internal Medicine and Medical Specialties (POMISE), Azienda Ospedaliera Universitaria Policlinico ‘‘Paolo Giaccone’’, University of Palermo, via del Vespro 129, Palermo 90127, Palermo, Italy.*

**Keywords:** Audiological surveillance, Noise-induced hearing loss, Police officers, Systematic review

## Abstract

**Introduction:**

Noise-induced hearing loss (NIHL) is defined as the sensorineural hearing loss caused by acute acoustic trauma or chronic exposure to high-intensity noises. Exposure to noises can lead to irreversible damage to the inner ear and, consequently, to a permanent shift of the hearing threshold. Police officers are particularly at risk of acute or chronic hearing damages. The aim of this study is to evaluate the hearing loss of police officers in relation to the occupational risk factors and clinical-anamnestic characteristics by collecting and analyzing existing data and evidence available in public databases.

**Materials and Methods:**

A systematic review was conducted according to the Preferred Reporting Items for Systematic Reviews and Meta-analyses group (PRISMA). Studies were included if they met inclusion and exclusion criteria. Study selection, data extraction, and quality assessment were conducted independently by two researchers.

**Results:**

Our initial literature search yielded 29 peer-reviewed articles. Out of 29 papers, only 10 were included in the review, after inclusion and exclusion criteria were applied the.

**Conclusions:**

Hypertension, smoking and alcohol intake significantly affect hearing performance. In addition, a history of acoustic trauma, use of ototoxic drugs, exposure to noise in leisure-time activities and failure to use ear protectors are often found in a fair number of subjects. NIHL is also related to the age of the subjects as well as the extent and duration of noise exposure. Furthermore, NIHL is also influenced by shooting practice sessions police officers are required to undertake as well as by the chronic exposure to traffic noise, especially in motorcycle police officers.

## Introduction

The World Health Organization (WHO) has stated that approximately 466 million people worldwide have disabling hearing loss and that this number will increase to approximately 900 million people in 2050, affecting more than 5% of the world's population (1). Moreover, hearing loss without recognized cause affects the economic system for about 750 billion dollars (2). 

The Ministry of Health reported that in 2019 in Italy there were about 7 million people suffering from hearing loss, corresponding to about 11.7% of the population throughout the national territory (3). 

The WHO states that the most common causes of hearing loss worldwide include genetic causes, birth complications, infectious diseases, ear infections, the use of ototoxic drugs and aging (2). In the United States, age, prolonged exposure to noise, ear infections, belonging to specific ethnic groups, employment status are the main causes of hearing loss (4). 

Noise-induced hearing loss (NIHL) represents sensorineural hearing loss caused by acute acoustic trauma or chronic exposure to high-intensity noise (5). 

Today many people work in particularly noisy environments because of their profession: bus drivers, airplane pilots, military personnel and in general all people who work in outdoor environments (6-9). 

Exposure to noise can lead to a temporary shift in the hearing threshold on audiometry which would resolve within approximately two weeks; a continuous exposure to noise could instead lead to irreversible damage to the inner ear and, consequently, to a permanent shift of the hearing threshold (10,11). In subjects exposed to noise there is also an alteration in the ABR test with impaired nerve conduction to the higher centers (12). In addition, it has been shown that subjects chronically exposed to noise show an alteration of the vestibular evoked myogenic potential (VEMPs) in response to cochlear or saccular damage caused by the noise itself (13,14). 

In addition to the above-mentioned professional categories police officers are also at risk, of acute or chronic hearing damages. Some of them are particularly exposed sources of noise, such as those working carried in the dog units and on duty in the streets and circulating in the streets with motorcycles (15,16). Furthermore, all officers are exposed to impulsive noise due to the gunshots during training sessions at the shooting range. However, to be protected against NIHL, external or internal, which ear protectors are used during the training sessions (17,18). The effect of impulsive noise due to gunshots has also been evaluated in the military environment where soldiers are more frequently exposed to this type of noise. In this context, it was found that the protection offered by well-tailored in-ear inserts and external headphones, can maximize protection from external noise (19). 

We conducted a systematic review aiming to evaluate the hearing loss of police officers in relation to the occupational risk factors and clinical-anamnestic characteristics.

## Materials and Methods

This study was conducted according to the Preferred Reporting Items for Systematic Reviews and Meta-Analyses group (PRISMA) by selecting data using the PRISMA NMA Checklist (www. prisma- statement. org/ extensions/networkmetaanalysis.aspx).

A systematic review was performed of public databases (PubMed, Medline, EMBASE, the Cochrane library and Google Scholar) by using a search strategy that included five keywords: noise AND induced AND hearing AND loss AND policemen. We included all the studies regardless publication date or publication status until 28 February 2021. Articles published in languages other than English were excluded. Studies were included if they met the following criteria: 

1) type of participants: studies on police officers of all ages and ethnicity.

2) type of clinical-instrumental assessment: studies with an analysis of the auditory assessment (audiometry) of patients in relation to the hearing-loss risk factors and characteristics.

3) types of study: randomized controlled trials (RCT), nonrandomized controlled trials (NRCT), cohort studies, before-and-after studies (including before-and-after comparison case studies), case-control studies.

Studies with the below features were excluded:

1) type of participants: patients who do not work as police officers.

2) type of clinical-instrumental assessment: studies in which patients underwent to clinical and instrumental assessment other than the ones listed in the inclusion criteria.

3) types of study: case reports, narrative reviews.

Data were extracted by two researchers independently; any disagreement about data extraction was discussed until a consensus was reached. The results of the systematic review were synthetized, classified and analyzed according to different individual variables, the study characteristics and results. The individual variables considered are the following: NIHL, noise exposure in leisure time, a history of occupational noise exposure, acoustic trauma, a family history of hearing loss, use of ototoxic drugs or exposure to chemicals, use of ear protectors, a history of ENT and/or non-ENT diseases, a history of upper airways diseases, pre-existing condition of hearing loss, transmission-type hearing loss, smoking, alcohol abuse, diabetes, hypertension, hypercholestero- lemia, fullness, tinnitus, dizziness.

The articles selected after the primary search were included/excluded by screening the title and abstract of each study and applying the inclusion/exclusion criteria. We removed duplicates from the primary search. We confirmed the remaining studies and validated them.

## Results

Our initial literature search yielded 29 peer-reviewed articles. After applying the inclusion and exclusion criteria, we retained 10 studies to be included in the systematic review. We then extracted the data for each individual variable from the result section of the selected articles.

Three studies (9,17,20). Had only male participants; in four studies (16,21-23) male and female populations were included, while in the remaining three studies the sex of the participants was not specified (24-26). NIHL was found in five studies and in 641 subjects in total (21,22,24-26). Noise exposure in leisure time was found in three studies corresponding to a total of 249 subjects (9,16,23). A history of occupational noise exposure was found in only one study (total number of subjects = 225) (16). An acoustic trauma was reported in two studies for a total of 27 subjects (16,22). A family history of hearing loss was found in three studies for a total of 76 subjects (9,22,23). The use of ototoxic drugs or exposure to chemicals were highlighted in three studies for a total of 49 subjects (9,22,23). The use of ear protectors was found in two studies involving a total of 239 subjects (21,23). Only one study, for a total 262 subjects, reported anamnestic history of non-ENT diseases ; the same study also highlighted that67 subjects had a positive medical history for ENT diseases (16). A history of ear disease was found in one study only (total number of subjects = 107) (9). A history of upper airways diseases was highlighted in one study and affecting 85 subjects (9). A pre-existing condition of hearing loss was found in one study, affecting 2 subjects (23), while evidence of transmission-type hearing loss was highlighted in two studies for a total of 62 subjects (9,26). 

Smoking was found in four studies for a total of 751 subjects (16,21,22,26). Alcohol intake was found in two studies for a total of 98 subjects (21,22). Diabetes was found in three studies, affecting a total of 62 subjects (21,23,26). Hypertension was found in three studies, for a total of 44 subjects (21,23,26). Hypercholesterolemia was found in only one study, affecting138 subjects (21). Ear fullness was found in only one study (total number of subjects = 39) (22). Tinnitus was reported in five studies with a total of 160 subjects (17,20,22,23,25). Dizziness was found in one subject in only one study (17). None of the articles included in the final review evaluated a specific firearm.

## Discussion

NIHL is defined as a sensorineural hearing loss due to exposure to acute or chronic high-intensity noises. Audiometry shows a hearing loss at high frequencies (3000 Hz to 6000 Hz and, in most of the cases, at 4000 Hz), which defines the hearing loss typical of this hearing profile.

Police officers are subjected to various occupational risks, injuries and diseases of various kinds (27). 

The studies included in this review analysed the hearing loss of police officers and the potential impact of noises they are exposed to, due to their occupation, in relation to different clinical-anamnestic characteristics.

Some of these studies confirmed a varying degree of NIHL in many subjects.

In particular, Win et al. evaluated the hearing performance of 365 police officers. The prevalence of NIHL was 34.2%, with a higher prevalence in males (37.7%), compared to females (23.9%) (21). A similar study was conducted by Lesage et al. on 1692 subjects, which included 887 police officers (including 33 motorcycle police officers) and 805 civil servants. Twenty-eight percent of the agents had NIHL, while in the civil servants’ group only 16% had hearing loss (16). Shrestha et al conducted a similar study on 110 police officers, of which 66.4% had noise-induced hearing loss, with bilateral involvement in 45 cases (40.9%) and unilateral involvement in 28 cases (25.4%) (22). 

Chauhan et al evaluated 88 traffic police officers by using smartphone-based audiometric testing (software Hearing Test TM). Among the participants, 80 subjects had NIHL (24 with mild hearing loss, 42 with moderate hearing loss, and 14 with severe hearing loss) (24). Singh et al. conducted a cross-sectional study on traffic police officers to assess the presence of NIHL and found that, out of a total of 421 employees examined, 342 subjects (81.2%) had a threshold (25). Rajenderkumar et al. conducted a study to specifically evaluate the traffic noise produced by vehicles during rush hours. They compared the noise levels registered in different hours of the day, based on the increase in the density of vehicles in the city. A sample of 100 traffic police officers was also recruited to evaluate the possible hearing loss induced by traffic noise. The results of their study showed that 21 out of 100 traffic police showed typical NIHL with a drop at 4KHz (26). Individual variables of the populations of the selected studies are summarized in Table 1.

**Table 1:** Individual variables of populations of the selected studies

Study	Study Population	NIHL	Leisure-Time Noise Exposure	Acute Noise Trauma History	HL Family History	Ototoxic Drugs History / History of Exposure to Chemicals	Use of Ear Defenders	Conductive HL
**Win et al.**	365	125	NA	NA	NA	NA	235	NA
**Caciari et al.**	357	NA	153	NA	29	33	NA	55
**Lesage et al.**	1692	NA	79	19	NA	NA	NA	NA
**Shrestha et al.**	110	73	NA	8	38	1	NA	NA
**Chauhan et al.**	88	80	NA	NA	NA	NA	NA	NA
**Malowski et al.**	30	NA	17	NA	9	15	4	NA
**Gupta et al.**	90	NA	NA	NA	NA	NA	0	NA
**Singh et al.**	421	342	NA	NA	NA	NA	NA	NA
**Rajenderkumar et al.**	100	21	NA	NA	NA	NA	NA	7
**Wu et al.**	20	NA	NA	NA	NA	NA	NA	NA
**Total**	3273	641	249	27	76	49	239	62

NA: not available

In one study, Win et al. found that participants had mild NIHL in 93% of the cases, moderate and severe NIHL in 3.5% of the cases (21). The study of Shrestha et al. found that 57 participants (51.8%), had mild hearing loss, 15 subjects (13.6%) had moderate hearing loss and only one subject (0.9%) had severe hearing loss (22). The data confirm that the professional category of police officers is at higher risk of developing NIHL, compared to the general population, depending on the extend of exposure (21,22,24-26). This is also confirmed by the finding of the typical pattern of hearing loss at the 4000 Hz frequency reported in some studies (16,22-24). These data are in line with the literature (15,28-31). The data extracted from the studies included in this review also show how the degree of hearing loss detected by the audiometric tests of the various study participants is variable and, in most cases, is a mild-moderate degree (WHO) (21,22), as confirmed by other studies (16,24,25,28). The clinical features of hearing loss for each study are shown in Table 2.

**Table2:** Degree of hearing loss according to the WHO

Study	Mild HL	Moderate HL	Severe HL	Profound HL
**Win et al.**	53	2	2	0
**Caciari et al.**	NA	NA	NA	NA
**Lesage et al.**	NA	NA	NA	NA
**Shrestha et al.**	57	15	1	NA
**Chauhan et al.**	NA	NA	NA	NA
**Malowski et al.**	NA	NA	NA	NA
**Gupta et al.**	NA	NA	NA	NA
**Singh et al.**	NA	NA	NA	NA
**Rajenderkumar et al.**	NA	NA	NA	NA
**Wu et al.**	NA	NA	NA	NA
**Total**	110	17	3	0

NA: not available

Win et al. confirmed a significant association between NIHL and hypertension (odds ratio 3.3; P <0.001) and, overall, how other variables (male sex, diabetes, longer service life) may contribute significantly to NIHL (21). In one study, Lesage et al. found 516 smokers (30.5%) with NIHL, of which 303 subjects belonged to the study group and 343 subjects to the control group. (16). Instead, Shrestha et al confirmed that alcohol intake and smoking habits have a strong association with noise-induced hearing loss (p value = 0.00). The odds ratio with 95% confidence interval was 4,481 (1,925-10,432) and 6,578 (2,306-18,764), respectively (22). Malowski et al. found two subjects with diabetes and six subjects with hypertension among the participants of their study (p <0.05) (23). Rajenderkumar et al. confirmed medical history of smoking, diabetes and hypertension as risk factors for NIHL. Of the total number of participants, 5% of them suffered from hypertension, 2% were diabetic and 31% were smokers (26). Therefore, a direct correlation results between the pre-existence of certain pathological conditions or the presence of certain risk factors and noise-induced hearing loss (16,21-23,26). Hypertension and smoking are the most important risk factors that several studies link to NIHL (32-34). Table 3 shows the data obtained from an analysis of the most important risk factors of the subjects.

**Table 3:** Risk factors

Study	Tuxedo	Alcohol Consumption	Diabetes Mellitus	Hypertension	Hypercholesterolaemia
**Win et al.**	162	28	58	33	138
**Caciari et al.**	NA	NA	NA	NA	NA
**Lesage et al.**	516	NA	NA	NA	NA
**Shrestha et al.**	42	70	NA	NA	NA
**Chauhan et al.**	NA	NA	NA	NA	NA
**Malowski et al.**	NA	NA	2	6	NA
**Gupta et al.**	NA	NA	NA	NA	NA
**Singh et al.**	NA	NA	NA	NA	NA
**Rajenderkumar et al.**	31	NA	2	5	NA
**Wu et al.**	NA	NA	NA	NA	NA
**Total**	751	98	62	44	138

NA: not available

In one study, Shrestha et al. found that 26 participants (23.6%) had tinnitus and 39 (35.5%) had “blocked sensation” in the ear (22). Malowski et al. reported 11 cases (36.7%) of tinnitus in the two groups of subjects studied (23). Gupta et al found that 68 subjects in their study had tinnitus, of which 55 (61%) had work-related tinnitus (20). Singh et al found 43 individuals who reported subjective tinnitus. In addition, a large number of subjects reported allergic rhinitis, while other subjects experienced other effects such as sleep deprivation and occasional headache (25). In a 10-year longitudinal study, Wu et al. reported a record of tinnitus and dizziness. 

In particular, after the first study session, nine subjects (45%) experienced tinnitus, but none of them complained dizziness or vertigo. However, in the second phase of the study10 years after the first observation, three subjects (25%) reported tinnitus and only 1 subject (8%) had episodes of dizziness without rotatory vertigo (17). These data confirm that tinnitus is the most reported symptom by patients with NIHL (17,20,22,23,25). 

The literature shows how this symptom is often reported by those suffering from hearing loss associated with inner ear disorders (35,36). Auditory and vestibular symptoms are reported in table 4.

**Table 4:** Symptoms

Study	Fullness	Tinnitus	Dizziness
**Win et al.**	NA	NA	NA
**Caciari et al.**	NA	NA	NA
**Lesage et al.**	NA	NA	NA
**Shrestha et al.**	39	26	NA
**Chauhan et al.**	NA	NA	NA
**Malowski et al.**	NA	11	NA
**Gupta et al.**	NA	68	NA
**Singh et al.**	NA	43	NA
**Rajenderkumar et al.**	NA	NA	NA
**Wu et al.**	NA	12	1
**Total**	39	160	1

*NA: not available*

## Conclusions

NIHL is a pathological condition that affects several occupational categories. This review highlights the impact that the environmental or occupational noise to which police officers are exposed to, can have on the function of the inner ear leading to significant alterations and resulting in transient or permanent hearing loss.

There are many factors that can lead to this condition: hypertension, smoking and alcohol intake significantly affect hearing performance. In addition, a history of acoustic trauma, the use of ototoxic drugs, exposure to noise during leisure time and the failure to use ear protectors are often found in a fair number of subjects.

NIHL is also related to the age of the subjects as well as the extent and duration of noise exposure. Furthermore, hearing loss is influenced by shooting practice sessions police officers are required to undertake as well as by the chronic environmental exposure to traffic noise, especially in motorcycle police officers.

A conscious use of ear protectors, both internal inserts and external headphones, prevention and education programs for police personnel as well as close audiological and occupational clinical surveillance of these subjects, could contribute to greatly reduce this unresolved problem strongly impacting on the health of police officers.

## Conflict of interest:

All authors have no conflict of interest to declare.

## Funding:

This research did not receive any specific grant from funding agencies in the public, commercial, or not-for-profit sectors. 
